# Calibrating your own fears: Feasibility of a remote fear conditioning paradigm with semi-subjective stimulus calibration and differences in fear learning

**DOI:** 10.3758/s13420-022-00545-1

**Published:** 2022-10-14

**Authors:** Frederic Berg, Jürgen Margraf, André Wannemüller

**Affiliations:** Mental Health Research and Treatment Center, Ruhr-Universität Bochum, Massenbergstraße 9, 44787 Bochum, Germany

**Keywords:** Fear conditioning, Stimulus calibration, Remote study, Fear generalization, Feasibility, Avoidance

## Abstract

Fear conditioning studies have occurred mostly in the laboratory, but recently researchers have started to adapt fear conditioning procedures for remote application. Standardization of aversive stimulus material not causing unnecessarily strong discomfort remains an issue especially relevant to research without experimental supervision. The present study introduces a novel semi-subjective method to calibrate aversive sounds in a remotely conducted fear conditioning paradigm. To demonstrate feasibility and proof of concept, 165 participants completed the paradigm, calibrating the loudness of an aversive sound without the guidance of an experimental instructor. This study also aimed to replicate existing findings of participant groups that differed in their early CS-UCS contingency awareness. Participants were classified as *Accurate* (UCS more likely after the CS+ than CS–), *Poor* (UCS more likely after the CS- than CS+, or UCS unlikely after either CS), and *Threat Biased* (UCS equally likely after the CS+ and CS–). Results indicated both the feasibility and efficacy of the paradigm, with participants showing typical patterns of fear learning. *Threat Biased* participants showed significantly higher uncertainty towards safety signals. There were no differences between the groups in terms of personality traits, thus questioning whether these attributes mediate differences in fear learning and the emergence of anxiety disorders. Using semi-subjective sound calibration appears to be functional, and future studies may consider implementing the new method when remotely administering fear conditioning paradigms.

The study of clinical phenomena such as phobias or anxiety disorders uses fear conditioning paradigms to explore the mechanisms which lead to and uphold psychopathology. The term *fear learning* covers various processes associated with the emergence of fear, such as *acquisition* or *safety-signal-learning*. Research addressing fear learning has been informed by fear conditioning experiments for decades, dating back to the *Little Albert* study by Watson & Rayner (1920), as well as early animal research conducted by Pavlov (1927). Since then, experimental methodology has advanced substantially (e.g., Lonsdorf et al., 2017; Ryan, Zimmer-Gembeck, Neumann, & Waters, 2019) and fear conditioning paradigms have been widely applied to study differences in fear learning, in both clinical and non-clinical samples (Duits et al., 2015).

By and large, these studies have used the same experimental procedures to unravel the various components of the conditioning process. Over time, participants acquire a fear response towards a formerly neutral cue (*conditioned stimulus*, *CS+*) that correlates with the onset of an aversive stimulus (*unconditioned stimulus*, *UCS*). Aversive stimuli have consisted of mild electroshocks or unpleasant loud sounds (Lonsdorf et al., 2017). In *differential fear conditioning paradigms*, a second neutral stimulus is introduced, which is never followed by an aversive stimulus. Over trials, this stimulus becomes a safety signal (*CS–*). By varying the components of the procedure (e.g., the type of CS and UCS, the CS-UCS contingency), the experimenter may examine the different facets of fear learning (Lonsdorf et al., 2017) and thereby better understand anxiety- or phobia-related pathologies (Vervliet & Raes, 2013; Dymond, Dunsmoor, Vervliet, Roche, & Hermans, 2015).

In an attempt to mitigate the problem of small sample sizes and thus often underpowered statistical analyses (Duits et al., 2015; Lonsdorf & Merz, 2017), researchers are starting to move away from the traditional approach of examining participants individually in the laboratory. The first steps to improve efficiency have been made by introducing fear conditioning paradigms into a large-group context, in which several participants go through the procedure simultaneously (Wannemueller et al., 2018). In 2019, Purves et al. introduced an app-based fear conditioning paradigm (Fear Learning and Anxiety Response, *FLARe*), which proved to be as effective in eliciting fear conditioning responses as the laboratory procedure. They concluded that this more flexible and cost-efficient method may prove useful for the study of subjective self-report data, albeit not able to assess physiological measures, which are currently limited to the laboratory setting. Recently, McGregor et al. (2021) used the FLARe app in a larger study (*N* = 1146) and showed that anxious participants had higher UCS expectancies after the CS- than non-anxious participants. Even though the authors discussed the limitations of not having direct control over the participant’s behavior, they concluded that the FLARe app allowed them to carry out the largest human fear conditioning study up until that moment.

In the same spirit, this study aims to facilitate conditioning research by providing a differential fear conditioning paradigm that should be (a) easy to conduct using the participants’ home computers, (b) safe in terms of health concerns, and (c) ensuring sufficient standardization even without an instructor. Considering these factors poses a strong challenge in terms of stimulus calibration, particularly when administering aversive stimuli, which, following the design used by Wannemueller et al. (2018), consist of aversive noises. To elicit a sufficiently strong response, unconditioned stimuli need to be intense enough to cause discomfort (Ryan et al., 2019). However, presenting unpleasantly loud sounds as unconditioned stimuli, when not properly calibrated before the first trial, might cause some participants significant and unnecessarily strong discomfort.

Thus, the central element of this adaptation is the unique volume calibration of an aversive sound that will serve as the UCS. In Purves et al. (2019), participants used their smartphones at maximum volume as a reference for the UCS intensity, whereas this study aims to combine an objective as well as a subjective calibration of the stimulus material, trying to minimize differences in the devices (i.e., PC, smartphone and earphones) used by the participant. This approach addresses several aspects considered advantageous regarding UCS calibration in fear conditioning paradigms, such as letting participants calibrate the stimulus themselves with respect to their personal threshold of pain (Lonsdorf et al., 2017; Merz & Lonsdorf, 2020) while maintaining a high level of standardization. We aim to demonstrate the feasibility of the paradigm as well as the functionality of this new approach to calibrating the volume of aversive sounds.

Differences in fear acquisition and extinction between groups of highly anxious individuals (or individuals suffering from diagnosed anxiety disorders) and healthy controls have been demonstrated in various aspects of the fear conditioning process, i.e., the general intensity of responding (e.g., Dvir, Horovitz, Aderka, & Shechner, 2019), pronounced discrimination between conditioned stimuli (e.g., Sjouwerman, Scharfenort, & Lonsdorf, 2020), attenuated extinction (e.g., McGregor et al., 2021), fear generalization (e.g., Stegmann et al., 2019; Lissek et al., 2014), and safety signal learning.

In light of fear conditioning research being employed as a translational model for psychopathology, discussion remains of whether temperamental or personality traits, as estimated by various psychometric measures, mediate existing differences in fear learning between patients and healthy controls. While differences in fear learning have been demonstrated between patients who typically score high on anxiety-related measures and healthy controls (Duits et al., 2015), predicting differences from temperamental variations alone remains largely inconclusive (Beckers, Krypotos, Boddez, Effting, & Kindt, 2013; Lonsdorf & Merz, 2017).

*Trait anxiety*, *neuroticism* or *anxiety sensitivity* represent possible candidates as they have been shown to be associated with anxiety disorders (Hur, Stockbridge, Fox, & Shackman, 2019) as well as with impaired safety signal learning (Gazendam, Kamphuis, & Kindt, 2013) and greater difficulty to correctly identify contextual safety cues in laboratory-based fear conditioning paradigms (Haaker et al., 2015). Nevertheless, the evidence is comparatively sparse (Lonsdorf & Merz, 2017) and it remains largely unclear if and to what extent the mechanisms of fear learning can be explained by differences in temperamental traits which we investigated in this study.

This study had two aims. The first aim was to provide a proof of concept for the introduced method of UCS calibration. To this end, various measures were employed, which included questioning participants about their experience with the procedure, the given instructions, and possible difficulties they may have faced while completing the paradigm. Of particular interest were questions of whether participants were able to complete the procedure without the presence of an instructor and whether they could successfully calibrate the UCS on their own, relying only on the written instruction provided. We hypothesized that, with the new calibration method, the conditioning procedure would engender the typical fear acquisition and extinction patterns (i.e., increase in fear towards the CS+ during the acquisition phase and its decrease during the extinction phase) as well as safety signal learning (i.e., decrease in fear towards the CS– over trials).

The second aim of this study was to replicate Wannemueller et al.’s (2018) findings concerning the participants’ awareness of the CS-UCS contingency. Using a clinical sample in a laboratory setting, the authors identified three groups: *Accurate*, participants who stated correctly that the UCS was more likely after the CS+ than the CS–, *Poor*, participants who stated that the UCS was more likely after the CS– than CS+ or that the UCS was unlikely after either CS, and *Threat Biased*, participants who stated that the UCS was as likely after the CS+ as the CS–. In the present study we asked whether a non-clinical sample exposed to a conditioning procedure with the self-calibration UCS would reveal the same groups. Of additional interest was whether the non-clinical *Threat Biased* group would also show, as its clinical counterpart did, overgeneralization of fear and impaired safety signal learning (i.e., a tendency to generalize the fear acquired to the CS+ to a cue that should actually be regarded as a safety signal; (Stegmann et al., 2019)), perhaps accompanied by significantly higher levels of trait anxiety, neuroticism, and anxiety-sensitivity than the other two groups. If so, the results would further attest to the relevance of overgeneralization of fear and impaired safety signal learning as possible risk factors for the development of pathological fears and anxiety disorders. They would also show the ecological validity of our instructor-free adaptation of Wannemueller et al.’s (2018) laboratory study.

## Methods

### Study sample

Following a priori power analyses (see Planned statistical analysis), we aimed to recruit a minimum of 158 participants. The study was distributed via the Ruhr University’s portal for advertising currently running studies and was primarily made up of psychology undergraduates. Various Facebook groups, in which study advertisement is permitted, were utilized to advertise the study to a broader spectrum of potential participants. To be included in the study, participants had to be at least 18 years of age, have a good grasp of the German language, no hearing impairment, and access to a computer with headphones and PowerPoint 2013 or later. Everyone taking part in the study was eligible to participate in a raffle for five €20 coupons. The necessary materials to take part in the study were sent out to 218 participants, of which 165 to date had completed the procedure and sent back their individual copies. One person was excluded from data analysis because they had always checked the middle item in all questionnaires and thus did not show any variance.

### Procedures

**Fig. 1:**
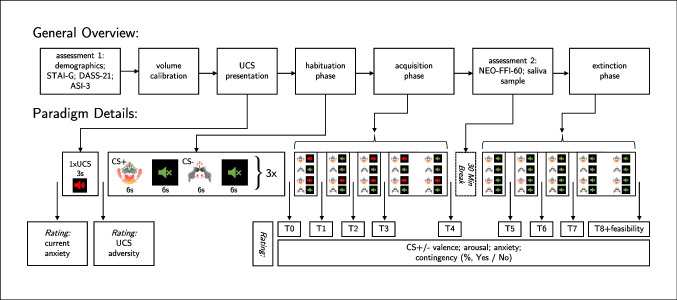
Experimental phases and events

Participants received a link to an online survey that asked them the contact information, used subsequently to mail the documents required for the study and the consent form. Next, participants received all questionnaires, instructions, and the declaration of consent via mail. The PowerPoint presentation, containing the fear conditioning paradigm, was sent out via e-mail. Participants were able to complete the study on their own, with only the provided instructions. Figure 1 illustrates the procedure and provides details on experimental phases and employed measures. Once finished, participants were asked to send all documents back to the investigator. All participants gave their informed written consent to take part in the study. The study received approval by the Ruhr University’s ethics committee (number 698) and was preregistered using the *AsPredicted* preregistration form on osf.io (Berg & Wannemüller, 2021).

#### Semi-subjective UCS calibration

To ensure the most optimal UCS salience, a semi-subjective stimulus calibration was developed for this study. The sound of a fork scratching over slate, as previously proposed by Neumann & Waters (2006) was used as the UCS. This sound has been widely used in other studies (e.g., Waters, Theresiana, Neumann, & Craske, 2017; Wannemueller et al., 2018) and is acknowledged as a suitable stimulus to be used in differential fear conditioning paradigms (Ryan et al., 2019).

Following Wannemueller et al. (2018), we aimed to present the aversive sound at 85 decibels (dB). To circumvent the problem of individual differences in participants’ system volume, a generic 1 s beeping sound (Freesound, 2014) was chosen as a test stimulus, which was presented to participants at two different levels of volume prior to starting the conditioning paradigm. Given that the decibel scale does not have a specific metric but rather works as a relational measure of two sound volumes (Gelfand, 2010), the audio software Audacity 2.4.2 (Audacity Team, 2021) was used to level two iterations of the beeping sound 85 dB apart from each other. The 85-dBs difference remains the same, irrespective of the actual volume the sounds are played at. Using software, the first (softer) beeping sound’s volume was prepared so that it could only be heard when having one’s system volume at close to 100%.

In calibration, participants were asked to adjust their headphones or system volume, to the point of barely being able to hear the softer beeping sound. Given that the absolute threshold of hearing is defined as 0 dB (Gelfand, 2010), the softer beeping sound was used as the reference volume for the second beeping sound. If participants correctly leveled their volume, the second louder beeping sound would play at 85 dB. Participants would then listen to the second beeping sound (approximately playing at 85 dB) and decide if its volume caused them ear pain. If so, participants were asked to lower their volume to the point of the sound being very uncomfortable while bearable. Participants could play both beeping sounds as often as they needed to achieve the correct calibration. Exact instructions may be viewed in the additional material provided in Appendix Section A. Using Audacity, the aversive UCS was set by us to be as loud as the louder of the two beeping sounds. This calibration method ensured, that even though the aversive UCS (fork scratching over slate) was never played to participants in the calibration phase, it would play at the desired volume (i.e., at approximately 85 dB) once the actual conditioning paradigm started (see Fig. 1, *UCS presentation*). Thus, the louder beeping sound served as a kind of proxy, allowing for volume adjustment without risking early habituation to the aversive UCS, had it been used for calibration purposes.

#### Differential fear conditioning paradigm

Following the semi-subjective stimulus calibration, participants were instructed to have the documents related to the experiment ready and to start the differential fear conditioning paradigm. From this point on, the PowerPoint presentation ran automatically. First, the UCS was presented unrelated to any CS, to establish baseline aversiveness of the utilized sound. Next, a *habituation* phase followed, in which both CSs (two Rorschach pictures) were presented in the absence of the UCS. The visual stimulus material and further details of the procedure may be viewed in Fig. 1. Both CSs alternated with a black screen, each presented for 6 s. In the subsequent acquisition phase, both CS+ and CS– were presented ten times in random order, with the CS+ preceding the UCS eight times, thus achieving an 80% contingency rate. In conjunction with the black screen, the UCS was played immediately after the CS+ offset.

After acquisition, participants were instructed to take a 30-min break in which they needed to fill out additional questionnaires, as well as give a saliva sample which will be used for future DNA analyses that were not part of this study. After the break, participants could proceed, and the presentation ran automatically again. In the extinction phase, both CS+ and CS– were presented ten times and were never reinforced.

### Measures

Participants were asked to provide information about sex, age, marital status, highest level of education (including the number of years spent in any sort of academic institution), and occupational status.

#### Measures regarding the feasibility of the study

Right after the last assessment of the CSs (see Fig. 1, *T8+feasibility*), participants were asked five questions, concerning the feasibility of the experiment. Clarity of the overall instructions, *How understandable did you find the instructions of the experiment?*, and clarity of the instructions regarding the stimulus calibration, *How understandable did you find the instructions for setting the volume?*, were rated on an 11-point Likert scale ranging from 0 = *very incomprehensible* to 10 = *very comprehensible*. Participants were also asked whether they felt like they were able to complete the experiment on their own with no instructor present. Two questions relating to the UCS followed: *In retrospect, how would you rate the loudness of the unpleasant sound? It should have been loud and unpleasant, but not painful.* rated on an 11-point Likert scale ranging from 0 = *The sound was too quiet* to 10 = *The sound was painfully loud*. Because we could not control for the possibility that participants would lower their system’s volume after hearing the aversive sound for the first time, they were asked: *Did you turn down your headphones after the first presentation of the unpleasant noise?*. Finally, participants had the opportunity to give recommendations or other thoughts they wished to share after having completed the experiment.

#### Measures regarding acquisition and extinction of fear

Prior to the first UCS presentation, participants were asked to estimate their current level of *state fear* on an 11-point Likert scale from 0 = *not at all fearful* to 10 = *very fearful*: *How fearful are you at this point?*. After a single presentation of the aversive sound, UCS aversiveness was assessed on the 9-point Self-Assessment Manikin (SAM) (Bradley & Lang, 1994) scale: *Please indicate how you felt about the sound you just heard.*. Subsequently, changes in elicited *subjective fear*, were assessed. Subjective fear was estimated by asking: *How fearful do you feel when you look at this picture?*, assessed on an 11-point Likert scale. In addition, participants were asked to state whether they expected the aversive sound to appear after each CS (*CS contingency [yes / no]–Do you think that after the picture the sound will be heard?*). Lastly, participants were asked to estimate the probability (*CS contingency [0–100 %]*) with which they believed the aversive sound appeared after each CS: *What do you think is the likelihood that this image will be followed by a sound?*. This measure was initially applied after the first acquisition phase (T1). The ratings were collected at nine time points (see *T0–T8* in Fig. 1).

#### Personality and anxiety-related measures

The State-Trait-Anxiety Inventory German version (STAI-G) estimates state- and trait anxiety and has been used extensively in clinical as well as non-clinical research (Spielberger, 1983; Laux & Spielberger, 2001). The measure shows high test–retest reliability (between *r* = .77 to *r* = .90) for trait anxiety (Laux & Spielberger, 2001). In the present sample, we found very good reliability indices in terms of internal consistency (*α* = .92). This analysis made use of the trait anxiety scale.

The German Depression, Anxiety and Stress Scale-21 (DASS-21) is used to assess a person’s strain caused by symptoms of depression, anxiety and stress (Lovibond & Lovibond, 1995; Nilges & Essau, 2015). The participants’ assessments should reflect their experience over the last 7 days. The DASS-21 includes items concerning autonomic arousal and skeletal muscle effects, thus considering the link between state anxiety and the acute bodily fear response. The DASS-21 anxiety subscale showed good internal consistency (*α* = .83).

The German Anxiety Sensitivity Index-3 (ASI-3) (Kemper, Ziegler, & Taylor, 2011; Taylor et al., 2007) measures the construct of anxiety sensitivity, which refers to how sensitive a person is to experiencing symptoms of anxiety (Reiss, Peterson, Gursky, & McNally, 1986). Internal consistency in the present sample was high (*α* = .89). In terms of validity, moderate-to-high correlations were observed for other measures of anxiety, depression and neuroticism (Kemper et al., 2011).

Lastly, the 60-item NEO-Five Factor Inventory (NEO-FFI-60) is used to measure an individual’s expression of the Big-Five personality traits (Gerhard, 1999; Costa & McCrae, 2008; Goldberg, 1990). Following the formulated hypotheses, the Neuroticism scale was employed for this analysis. Internal consistency was good (*α* = .87).

### Planned statistical analysis

Statistical analyses were carried out in *R for Windows* (Ver. 4.0.2; R Core Team, 2020). Descriptive statistics are provided for all employed measures, as well as data describing the sample. In order to provide the most transparent analysis in the context of testing the feasibility and efficacy of the newly employed stimulus calibration method, it was decided to include all participants in the analysis. Still, to remedy possible effects of outliers, Cook’s distance was calculated for each of the below described group comparison models. There were no outliers showing significant leverage, thus corroborating the decision of not excluding any participants. Null hypotheses were tested against a threshold of significance of *p *< .05. A priori power analyses were computed using G*Power (Ver. 3.1.9.4; Faul, Erdfelder, Lang, & Buchner, 2007).

#### Assessment of feasibility and proof of concept

Measures of feasibility were analyzed descriptively. We compared participants who had indicated turning down their headphones’ volume following the initial presentation of the aversive UCS to those who did not. Violations of the assumption of normality were addressed by computing non-parametric Mann–Whitney *U* tests, corresponding effect size was *r* (rank-biserial correlation). Following the analysis by Wannemueller et al. (2018), three distinct groups of threat contingency awareness were determined by analyzing participants’ replies to the dichotomous forced choice CS-UCS contingency items after early acquisition (i.e., at T1). Participants who expected the UCS to follow the CS+, but not the CS–, were labeled *Accurate*, while individuals who expected the UCS to follow neither CS or only the CS– were labeled *Poor*. In case an individual expected the UCS to follow both CS+ and CS–, they were identified as *Threat Biased*. Next, we compared the distribution of participants across the three groups with the distribution obtained by Wannemueller et al. (2018).

To determine whether the acquisition and subsequent extinction of fear to the CS+ took place, as well as the identification of CS– as a safety signal, we performed two mixed ANOVAs (type III, expecting significant interactions) with the *subjective fear* ratings of each stimulus as the dependent variable (DV). Measurements from T0–T8, were included (within-subjects variable, henceforth referred to as *Time of Measurement*). Participants who stated that they had lowered their headphones’ volume after the initial UCS presentation were compared to participants who did not (between-subjects variable, in further course referred to as *Volume Manipulation*). This was done to investigate whether fear conditioning took place in spite of this deviation from the intended procedure (i.e., changing system volume after the actual calibration phase). Success of acquisition, extinction and safety signal learning were analyzed by comparing the overall means of these two groups.

Two other mixed ANOVAs compared the subjective fear ratings of CS+ and CS– in the three previously described contingency awareness groups (between-subjects variable), and across T0 to T8 (within-subjects variable).

Lastly, two mixed ANOVAs compared the participants’ CS-UCS contingency ratings of the CS+ and CS– (DV) across the three contingency awareness groups and the eight moments (T1 - T8), for a total of six mixed-design ANOVAs.

In case of significant results, post hoc pairwise comparisons of means were carried out. Further, in case of significant interactions, differences between contingency groups and also regarding Volume Manipulation, were investigated by pairwise mean comparisons. The Tukey method was used to account for multiple testing.

Following the proposed hypotheses, post hoc pairwise comparisons were carried out with the aim of reporting contrasts that illustrate successful Acquisition of fear towards the CS+ (i.e., T0 vs. T4), subsequent Extinction of that fear (i.e., T4 vs. T8) and Safety Signal Learning (i.e., fear at T0 vs. T8 regarding the CS–). Contrasts within points in time were carried out in case of significant interactions to check whether there were significant differences in fear learning between contingency awareness groups or between participants who lowered their system’s volume following the initial UCS presentation and those who did not. Of interest would be a possible increase in fear towards the CS– from T0 to T1 which might serve as evidence of fear generalization. Since *Threat Biased* participants were suspected of exhibiting higher levels of trait anxiety, we expected this group to show an increase in fear, as well as greater uncertainty towards the safety signal.

Calculated effect size for post hoc contrasts was Cohen’s *d*. In terms of statistical assumptions, all data were screened for additivity as well as tested for linearity and departures from normality and homogeneity of variances by visual inspection of respective plots. Departure from sphericity was tested by conducting Mauchly’s test and was subsequently accounted for by reporting Greenhouse-Geisser corrected *p* values. A priori power analyses were conducted, assuming medium effect sizes (*f* = .25), *α*-error probability of .05, moderate power (1 - *β* = .80) and following the recommendations provided by Bartlett (2021) correlation among repeated measures of 0.5 and a nonsphericity correction of *𝜖* = .15, which yielded a total necessary sample size of *N* = 69 for the mixed ANOVAs.

#### Hypotheses testing: Differences in fear learning and temperamental traits

The initial increase in fear towards the CS– was calculated by subtracting the baseline subjective fear towards the CS– from subjective fear towards the CS– following the first acquisition phase (CS–*fear*_*inc*_ = [*subjective fear* CS– at T1] – [*subjective fear* CS– at T0]). Violations of the assumptions of normality and or homogeneity of variances were remedied by calculating non-parametric Kruskal-Wallis tests instead of one-way ANOVAs. Significant differences between contingency groups were further investigated by computing Dunn’s tests, adjusted with the Bonferroni method. The reported effect size was *η*^2^. A priori power analyses were calculated assuming medium effect sizes (*f* = .25), which yielded a total sample size of *N* = 158.

## Results

### Sample characteristics

Total sample size was at *N* = 165, with a mean age of *M* = 23.68 (*SD* = 5.36). For further details please refer to Table 1.

### Feasibility of the paradigm

Table 2 summarizes the results on the four items that assessed the participants’ experience with the procedure, distinguishing the participants who manipulated the volume after the first UCS presentation from participants who did not. Scores for all feasibility items were high, indicating that the instructions were in general understandable and sufficient to complete the procedure without an instructor present. Participants who did not alter their volume reported a significantly higher estimation of how well they could orient themselves without an instructor present, *W* = 3365, *p* = .045, with an effect size of *r* = -.13. That same group reported a significantly lower loudness of the UCS, *W* = 2295, *p* = .027, with an effect size of *r* = -.15.

### Proof of concept

#### Acquisition and extinction of fear & safety signal learning

**Fig. 2:**
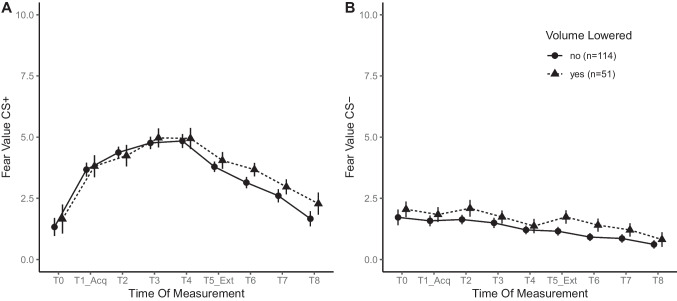
Fear values over time in individuals who manipulated their volume & those who did not. **A** and **B** show mean fear towards CS+/– over time, respectively, as a function of whether participants lowered their volume after the first UCS presentation. *Error bars* indicate SEM

All six mixed-design ANOVAs met the statistical assumptions (see Planned statistical analysis) except for sphericity. Hence, all *p* values presented below are corrected by the Greenhouse–Geisser procedure. Figure 2 illustrates the changes in mean fear values towards both CS+ and CS– over the course of the procedure, comparing participants by volume manipulation. For reported fear towards the CS+, the analysis revealed a significant effect of time of measurement, *F*(8, 1296) = 93.43, *p* < .001. There was no significant effect of volume manipulation (*p* = .481) nor of its interaction with time of measurement (*p* = .580).

Pairwise post hoc comparisons revealed an increase in fear towards the CS+ from T0 to T4 (1.49 vs. 4.90, *p *< .001, *d* = -1.07). Further, a decrease of fear from T4 to T8 (4.90 vs. 1.97, *p *< .001, *d* = .92) was observed. These two contrasts indicate successful acquisition and subsequent extinction of fear towards the CS+.

The analysis of reported fear towards the CS– revealed a significant effect of time of measurement, *F*(8, 1296) = 19.22, *p* < .001, but no significant effects of volume manipulation (*p* = .167) or its interaction with time of measurement (*p* = .758).

Pairwise post hoc comparisons revealed that fear towards the CS– decreased from T0 to T8 (1.89 vs. .72, *p *< .001, *d* = .51). The reduction in fear suggests that the CS– was correctly interpreted as a safety signal. Fear learning occurred similarly between participants who did and did not manipulate the sound volume following the initial UCS presentation.

**Table 1:** Sociodemographics and primary outcome descriptives of T1 contingency groups

	Total sample	Accurate	Poor	Threat biased	Group
	(*N* = 165)	(*n* = 122)	(*n* = 30)	(*n* = 13)	Comparisons
	(100.00 %)	(73.90 %)	(18.20 %)	(7.90 %)	*p* values^a^
Age	23.68 (5.36)	23.65 (5.09)^b^	23.23 (5.18)	25.17 (8.13)	.667
(min–max)	(18 – 46)	(18 – 46)	(19 – 37)	(19 – 43)	
Sex, *n (%)*					.003*
Female	130 (78.79 %)	101 (82.79 %)	17 (56.67 %)	12 (92.31 %)	
Male	32 (19.39 %)	20 (16.39 %)	12 (40.00 %)	0 (0.00 %)	
Diverse	1 (0.61 %)	1 (0.82 %)	0 (0.00 %)	0 (0.00 %)	
Missing information	2 (1.21 %)	0 (0.00 %)	1 (3.33 %)	1 (7.69 %)	
Marital status, *n (%)*					.540
Single	76 (46.06 %)	57 (46.72 %)	14 (46.67 %)	5 (38.46 %)	
In relationship	76 (46.06 %)	57 (46.72 %)	13 (43.33 %)	6 (46.15 %)	
Married	8 (4.85 %)	5 (4.10 %)	2 (6.67 %)	1 (7.69 %)	
Divorced	2 (1.21 %)	2 (1.64 %)	0 (0.00 %)	0 (0.00 %)	
Missing information	3 (1.82 %)	1 (0.82 %)	1 (3.33 %)	1 (7.69 %)	
Vocation, *n (%)*					.363
Student	145 (87.88 %)	108 (88.52 %)	26 (86.67 %)	11 (84.62 %)	
Student & Employee	4 (2.42 %)	4 (3.28 %)	0 (0.00 %)	0 (0.00 %)	
Employee	76 (7.27 %)	7 (5.74 %)	4 (13.33 %)	1 (7.69 %)	
Self-employed	2 (1.21 %)	2 (1.64 %)	0 (0.00 %)	0 (0.00 %)	
Unemployed	1 (0.61 %)	1 (0.82 %)	0 (0.00 %)	0 (0.00 %)	
Missing information	1 (0.61 %)	0 (0.00 %)	0 (0.00 %)	1 (7.69 %)	
Level of education, *n (%)*					.008*
Secondary school	2 (2.42 %)	2 (1.64 %)	2 (6.67 %)	0 (0.00 %)	
High school	139 (84.24 %)	107 (87.70 %)	23 (76.67 %)	9 (69.23 %)	
University degree	21 (12.73 %)	13 (10.66 %)	5 (16.67 %)	3 (23.08 %)	
Missing information	1 (0.61 %)	0 (0.00 %)	0 (0.00 %)	1 (7.69 %)	
Years in academic institutions	15.01 (3.18)	15.01 (3.29)	14.78 (2.68)	15.59 (3.40)	.584
(min–max)	(11.5 – 40)	(12 – 40)	(11.5 – 25)	(12.5 – 25)	
Temperamental measures					
STAI X2 Trait Anxiety	39.91 (10.48)	39.36 (10.15)	42.55 (11.72)	39.00 (10.38)	.290
DASS-21 Anxiety	2.88 (3.51)	2.62 (3.28)	3.87 (4.13)	3.08 (3.93)	.242
ASI-3 Anxiety Sensitivity	7.78 (4.69)	7.77 (4.67)	8.40 (5.06)	6.43 (3.90)	.515
NEO-FFI-60 Neuroticism	21.72 (8.36)	21.27 (8.25)	23.63 (9.20)	21.54 (7.25)	.383^c^
CS– *fear*_*inc*_	-0.16 (1.58)	-0.32 (1.57)	0.27 (1.31)	0.31 (2.06)	.049*
Baseline Fear T0	1.92 (2.06)	1.79 (2.05)	2.23 (1.94)	2.46 (2.47)	.266
UCS Aversiveness T0	6.74 (1.60)	6.92 (1.40)	6.10 (2.20)	6.62 (1.39)	.182
Volume manipulation					.505
No	114 (69.09 %)	82 (67.21 %)	21 (70.00 %)	11 (84.62 %)	
Yes	51 (30.91 %)	40 (32.79 %)	9 (30.00 %)	2 (15.38 %)	

^a^ The *asterisks* identify significant differences between the three groups according to Kruskal–Wallis or *χ*^2^-tests

^b^ Unless otherwise specified, each cell shows mean (standard deviation)

^c^ One-way ANOVA

**Table 2:** Feasibility outcomes of volume manipulation groups & total sample

	Total sample	Manipulation: *No*	Manipulation: *Yes*	Group
	(*N* = 165)	(*n* = 114)	(*n* = 51)	Comparisons
		(69.09 %)	(30.91 %)	*p* values^a^
Clarity of instructions	9.22 (1.38)	9.17 (1.57)	9.35 (0.80)	.730
Clarity of volume calibration	9.10 (1.58)	9.19 (1.58)	8.88 (1.57)	.131
Orienting without instructor	9.42 (0.97)	9.50 (0.95)	9.24 (0.99)	.045*
Loudness of UCS	6.39 (1.37)	6.23 (1.36)	6.76 (1.34)	.027*

Outcomes of feasibility related items, comparing participants who stated that they lowered their headphones’ volume after the initial presentation of the aversive UCS and participants who did not. Values presented are *M* = mean, *(SD)* = standard deviation.

^a^ The *asterisks* identify significant differences between the two groups according to Mann–Whitney *U* tests

### Differences in fear learning between contingency awareness groups & temperamental traits

#### CS-UCS contingency awareness groups at T1

The proportional distribution of participants across the three contingency awareness groups did not match the distribution obtained by Wannemueller et al. (2018). The present study found relatively more *Accurate* (73.90 vs. 62.0%) and fewer *Poor* (18.20 vs. 21.0%) and *Threat Biased* (7.90 vs. 17%) participants than the previous study. Table 1 details differences in demographics as well as temperamental and fear learning-related outcomes. The makeup of participants within each group significantly differed in terms of sex, *χ*^2^(6, 165) = 19.52, *p* = .003 and highest level of education, *χ*^2^(6, 165) = 17.14, *p* < .008.

There was a significant difference between the groups’ initial increase in fear towards the safety signal (CS–*fear*_*inc*_), *H*(2) = 6.05, *p* = .049, *η*^2^ = .025, with both *Poor* and *Threat Biased* groups showing an increase in fear, while the *Accurate* group showed a decrease. Post hoc Dunn’s tests revealed a significant difference when comparing *Accurate* and *Threat Biased* groups, however, this difference did not reach the threshold of significance when using Bonferroni’s correction for multiple comparisons, *p* = .129. Thus, it seems that *Threat Biased* participants did not exhibit fear generalization to the CS–.

#### Fear learning differences between contingency groups

**Fig. 3:**
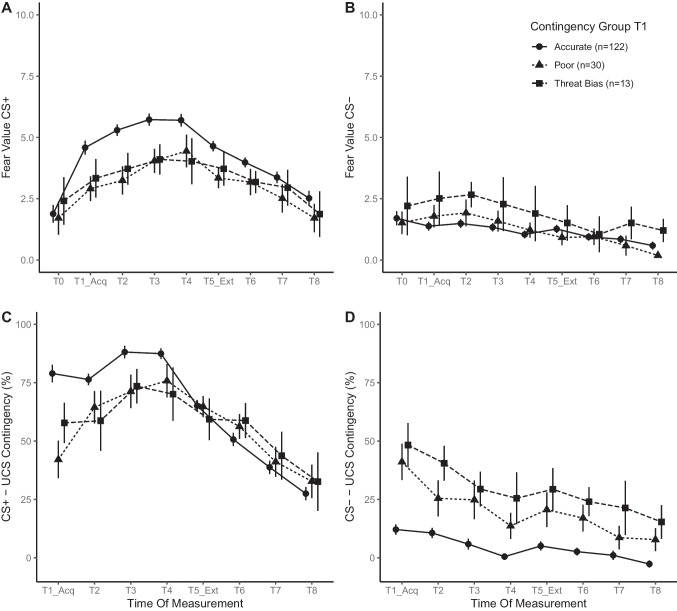
Fear values & contingency probability ratings over time by contingency awareness at T1. **A** and **B** present mean fear towards CS+/–, respectively, over time. **C** and **D** display probability ratings regarding the CS-UCS contingency over time. In all four panels contingency awareness groups identified at T1 are compared. *Error bars* indicate SEM

As the previously described analyses already illustrated the overall effects of acquisition, extinction and safety signal learning, the following two analyses focus on the main effects of the contingency awareness groups and its interaction with time of measurement. Figure 3 depicts differences in subjective fear (panels A and B) and contingency probability ratings (panels C and D) towards both CSs between contingency awareness groups.

##### Differences in subjective fear

For subjective fear towards the CS+, the analysis revealed a significant main effect for contingency awareness groups, *F*(2, 161) = 4.03, *p* = .020 and the interaction between time of measurement and contingency awareness groups, *F*(16, 1288) = 2.94, *p* = .005. Although the *Accurate* group did show the strongest fear response to the CS+ in acquisition, after correcting for multiple comparisons, pairwise post hoc comparisons did not show any meaningful significant differences between the groups within any of the nine points in time (T0 to T8). After the initial increase, fear towards the CS+ appears to have stabilized at comparable levels in all three groups. For fear towards the CS–, neither the main effect of contingency awareness groups (*p* = .262) nor the interaction between time of measurement and contingency awareness groups (*p* = .105) reached significance. There was no meaningful difference in safety signal learning.

##### Differences in contingency probability ratings

The overall probability with which participants expected the UCS to follow the CS+ changed over time, with the analysis revealing a significant main effect of time of measurement, *F*(7, 1134) = 78.71, *p *< .001, contingency awareness groups, *F*(2, 162) = 6.75, *p* = .002, and their interaction, *F*(14, 1134) = 11.75, *p *< .001.

Pairwise post hoc comparisons revealed a significant overall increase in the probability ratings from T1 to T4 (59.6 vs. 77.8, *p* < .001, *d* = -.41) and a significant decrease from T4 to T8 (77.8 vs. 30.9, *p *< .001, *d* = 1.06). At T1, group *Accurate* differed from the other two groups (*Poor*: 78.9 vs. 42.1, *p *< .001, *d* = .66; *Threat Biased*: 78.9 vs. 57.8, *p* = .035, *d* = .27).

*Poor* and *Threat Biased* groups did not differ from each other. At later time points, there were no significant differences.

The overall probability ratings of expecting the UCS to follow the safety signal (i.e., the CS–) changed over time. The analysis showed a significant main effect for both time of measurement, *F*(7, 1134) = 35.88, *p *< .001, and contingency awareness groups, *F*(2, 162) = 29.83, *p* < .001, as well as a significant interaction, *F*(14, 1134) = 3.96, *p* < .001.

Pairwise post hoc comparisons revealed a significant overall decrease in the estimated probability of the UCS following the CS– from T1 to T8 (33.81 vs. 6.82, *p* < .001, *d* = .78).

The *Threat Biased* group showed the highest probability ratings throughout the procedure. The differences between this group and the *Accurate* group reached significance at all but the last measurement (T8), with effect sizes ranging from small to medium effect sizes (all *d* values between, *d* = -.64 and *d* = -.32). The *Poor* group showed significantly higher estimates as well when compared to the *Accurate* group. This difference was not significant at T4, T7 and T8. Effect sizes were smaller, except for the first contrast (all *d* values between, *d* = -.74 and *d* = -.19). There were no significant differences between *Threat Biased* and *Poor* groups.

## Discussion

This study introduced a new, semi-subjective method of calibrating the aversive stimulus of a fear conditioning procedure to be applied remotely. It tested the method within a non-clinical sample of participants and compared its findings with those obtained previously within a clinical sample (Wannemueller et al., 2018). It also added to the existing but largely inconclusive literature on the association of temperamental factors and differences in fear learning.

### Feasibility and proof of concept of the paradigm

Feasibility was assessed by asking participants about their experience in going through the study without an experimental instructor. Ratings for all items were very high, with most participants indicating the highest score for all the measures assessing understandability of the instructions. These results suggest that, when provided with sufficiently detailed instructions and information, a differential fear conditioning paradigm may validly be carried out without an instructor. Being able to remotely employ these types of paradigms may increase considerably the access to and yield in participants, as seen most recently with the use of the FLARe app (McGregor et al., 2021; Purves et al., 2019). Additionally, in light of the current COVID-19 pandemic, these types of paradigms can be administered even if laboratories need to be closed or if participants cannot come to the research site.

Key to the newly adapted paradigm was the unique way in which the aversive unconditioned stimulus was calibrated. This novel method combined both an objective, as well as a subjective component of calibration. First, leveling the volume with respect to a softer test sound ensured the UCS volume to be very close to the aspired volume of 85 dB. Secondly, as recommended by Lonsdorf et al. (2017), allowing participants to adjust their headphones’ volume after hearing a test-sound that was as loud as the aversive UCS made it possible to include their individual pain tolerance. Because there was no instructor present, avoiding potential harm should be an important concern when thinking about adequate stimulus application. Britton et al. (2013) employed the sound of a human scream as the aversive UCS and saw a substantial drop out of participants from the first to the second day of measurement. Although only adolescents dropped out while adults did not, the result highlights the need for adequate stimulus selection and calibration. Also, future research may consider exploring differences between various types of aversive sounds, including highly aversive ones like human screams. Lastly, it needs to be emphasized that an increase of 10 dB leads a person to perceive the sound as approximately twice as loud (Gelfand, 2010), which means that even seemingly small deviations from the aspired value may lead to severe differences in perception.

Right after the initial UCS presentation, participants rated it as very unpleasant (see Table 2*, UCS Aversiveness T0*). In addition, when the participants were asked at the end of the experiment to tell whether they found the UCS to be either too silent or painfully loud, the mean score settled within the upper half of the provided scale. Because our goal was to present a UCS that was unpleasantly loud but not painful, the rating scores seem to express adequate stimulus calibration. Another feasibility item asked participants whether they had lowered the volume after hearing the UCS. Although approximately one third of the participants acknowledged lowering the volume, we found no significant differences between those participants and the remaining ones in the self-reported fear towards the CS+ and the CS- (see Fig. 2). This result shows that lowering the volume after the calibration phase, a divergence from the instructions, did not render the UCS too silent or insufficiently aversive. It may be speculated that the sound, as proposed by Neumann and Waters (2006), is by its nature aversive enough to sufficiently induce fear, even at lower volumes. In combination with the UCS played back via headphones, which may feel more threatening compared to loudspeakers, the procedure’s set-up seems capable of compensating for between-subjects variation in calibration.

Lowering the volume after the initial UCS presentation may also be seen as a form of avoidance, which is viewed as a key mechanism in the emergence and persistence of anxiety disorders (e.g., Pittig, Treanor, LeBeau, & Craske, 2018). Interestingly, there was no difference in fear towards either CS between these two groups of participants. Thus, lowering the volume which one could speculate represents partial avoidance, did not prevent these participants from showing the same emergence of fear towards the CS+. This finding is in line with research showing that the possibility to avoid protects an individual from the extinction of fear (e.g., Lovibond, Mitchell, Minard, Brady, & Menzies, 2009).

Research on avoidance behavior in fear conditioning paradigms has focused on avoidance as a central element of the procedure, deliberately giving participants the option to avoid an aversive event (e.g., Klein et al., 2021b; Klein, Berger, Vervliet, & Shechner, 2021a; Lemmens, Beckers, Dibbets, Kang, & Smeets, 2021). This study suggests paying attention to participants’ avoidance behavior as a control variable in paradigms where the researcher has no direct control over avoidance. Future studies may try to identify which factors influence a participant’s decision to engage in avoidance and consider including a way to assess avoidance behavior when employing aversive sounds as unconditioned stimuli. In a more sophisticated version of such a paradigm, the degree to which participants manipulate their device’s volume may even be used to quantify the extent of avoidance.

Considering the overall success of learning (i.e., acquisition and extinction of fear, as well as safety signal learning), the newly adapted paradigm appears to be functional. The analysis of self-reported fear over time revealed a significant increase in fear towards the CS+ until after the acquisition phase (T0–T4). Subsequently, there was a significant decrease of fear, indicating extinction of the previously acquired fear. Both comparisons showed large effect sizes, highlighting the efficacy of the newly adapted paradigm and calibration method. Safety signal learning was also observed, with self-reported fear scores dropping from T0 to T8. Considering the results of the previously discussed measures of feasibility, stimulus calibration and now of the fear conditioning process itself, it seems appropriate to conclude that the adapted paradigm, using a novel semi-subjective stimulus calibration, is functional. Future studies, especially app-based and remotely conducted fear conditioning paradigms, may consider implementing a similar type of calibration to further refine this new way of carrying out these types of paradigms.

### Differences in acquisition and extinction of fear and safety signal learning

In addition to the commonly employed evaluative measure of self-reported fear, UCS contingency probability was chosen as a second measure, reflecting the associative part of fear learning (Constantinou et al., 2021). Boddez et al. (2013) argued for the validity of UCS expectancy ratings as an important measure, informing about an individual’s estimation of risk probability, which is of great interest to the understanding of psychopathology.

In order to replicate the finding by Wannemueller et al. (2018) of distinct groups differing in their early CS-UCS contingency awareness, participants were grouped with respect to their responses to the dichotomous forced-choice item at T1 (i.e., after the first acquisition phase). This study did not find the same distribution of contingency awareness groups, with the *Threat Biased* group being smaller compared to the proportions reported by Wannemueller et al. (2018). It is important to note that their sample was comprised of patients diagnosed with various specific phobias. Thus, it may be the case that one would expect to see fewer *Threat Biased* individuals in a non-clinical sample.

Over the course of the procedure, there were no differences in self-reported fear towards either CS between the three contingency awareness groups. UCS expectancy towards the CS+ showed a similar pattern of fear learning with an increase in rated probability over the course of the acquisition and a subsequent decrease in rated probability until after extinction. After early acquisition (T1) the *Accurate* group reported greater certainty about the CS+ - UCS contingency when compared to the other two groups. A similar pattern was observed for the CS–, where throughout all times of measurement, the *Accurate* group correctly indicated a very low probability of the aversive sound to follow the CS–. In contrast, *Threat Biased* participants showed a pattern of stronger uncertainty towards the CS–. Up until the last point of measurement, this group showed the strongest uncertainty, estimating the contingency probability significantly higher than the *Accurate* group. This finding is in line with the observations by Wannemueller et al. (2018), who also found *Threat Biased* participants to show the strongest uncertainty towards the safety signal. It is of note, that there were no statistically significant differences in rated probability between the *Threat Biased* and the *Poor* group. Further, the initial increase in fear towards the safety signal as observed in the *Threat Biased* group was very small and did not reach significance after correcting for multiple comparisons. However, the initial uncertainty towards the relationship of both CSs and the aversive UCS, as operationalized by the responses on the forced-choice item at T1, seemed to persist throughout the whole procedure.

Upon further analysis, we found that by the end of the acquisition phase, at T4, of the *Threat Biased* participants, two still showed the same pattern and two others exhibited the *Poor* pattern. Hence, by the end of the experiment, most participants were *Accurate*, they knew the correct contingencies. But still, initially *Threat Biased* participants showed much stronger uncertainty towards the safety signal’s relationship to the UCS throughout the paradigm.

The disconnect between the two types of measurement (anxiety vs. contingency probability) may reflect different facets of the fear learning process. Continuous probability estimates may represent a kind of risk estimation (Lonsdorf et al., 2017), which is central to the understanding of anxiety disorders in which risk-overestimation is regularly observed (Hengen and Alpers, 2019). On the contrary, there were no significant differences in fear learning between the *Threat Biased* and the *Poor* group, and also no differences in temperamental outcomes. This begs the question of whether the *Threat Biased* group is in fact distinct from the *Poor* group or if, at least in non-clinical samples, both are merely expressions of participants’ misunderstanding of questions, not paying close attention to the task or just making careless mistakes. However, the results by Wannemueller et al. (2018) showing *Threat Biased* participants to be more likely to carry two *5-HTTLPR* S-alleles, a phenotype of the serotonin transporter linked polymorphic region, and them showing the greatest uncertainty towards safety cues, supports the notion of a distinct phenotype. Since data collection for this study included the collection of genetic material from participants, future analyses may further inform about the validity of the described contingency awareness groups. Future studies may also explore whether in threat-biased participants, induced fear towards conditioned stimuli extinguishes at different rates across longer time periods (e.g., Amd, Machado, de Oliveira, Passarelli, & de Rose, 2019).

### Differences in conditionability and temperamental traits

The analysis of temperamental measures did not reveal any statistically significant differences between contingency awareness groups (see Table 2). In 2013, Torrents-Rodas et al. could not find any differences across both physiological as well as verbal measures between high and low-anxious individuals, supporting the view that even though there are some studies suggesting an effect of trait anxiety, most studies report no effects (Lonsdorf & Merz, 2017). A similar conclusion can be drawn for trait neuroticism (e.g., Arnaudova, Krypotos, Effting, Kindt, & Beckers, 2017). It might also be the case that, in remotely conducted studies, contextual factors (e.g., time of day or the presence of others) may have variance-explaining effects that offset the effects of personality or temperamental traits. Moreover, the results of the present study do not support an association between anxiety sensitivity and effects of fear generalization.

### Limitations and clinical implications

Although the newly adapted fear conditioning paradigm had several advantages worth considering for future research, some remaining issues need to be addressed. Since no experimental instructor was present to guide participants and guarantee standardized UCS calibration, some error variance will inevitably be introduced into the analysis. However, the results demonstrate the efficacy of the adapted procedure which in turn could increase the number of possible participants while also providing a method that allows reliable and safe calibration of the aversive stimulus material.

An obvious drawback of this study was the lack of physiological measures which to date are only applicable in the laboratory setting. Since studies employing various methods of *ambulatory assessment* have increased in the past years (see Carpenter, Wycoff, & Trull, 2016; Trull & Ebner-Priemer, 2020 for recent reviews), it seems a logical consequence to implement these types of assessment instruments in remote fear-learning research. In the context of app-based solutions, adapting the paradigm in PowerPoint may have further restricted the sample to individuals who own a copy of PowerPoint, which may be common in university students but more uncommon in non-students.

Out of 218 registered participants, to date, only 165 copies were sent back, so it remains unclear whether the 53 remaining participants faced troubles that prevented them from successfully completing the procedure. Even though the overall sample size of 165 participants was fairly large, the size of the *Threat Biased* group was still very limited. To further explore the differences between students and non-students, future studies should try to include as many non-student participants into the analysis as possible. Previous research revealed that instructing participants about the CS-UCS contingency prior to fear learning changed outcomes. Mertens, Boddez, Krypotos, and Engelhard (2021) showed that formerly instructed participants showed increased differential responding towards the conditioned stimuli, thus providing contingency information can reduce the effects of falsely expecting an averse UCS to follow a safety signal. A similar effect may be the case for the responding of students versus non-students. Given the fact that this study was primarily distributed within a population of psychology undergraduates, it can be assumed that most participants had at least basic knowledge about differential fear conditioning paradigms. Even though exact contingencies and block protocols were not disclosed to the participants, their expertise could have changed their expectations of what to anticipate. However, it is important to point out only one non-student was classified as *Threat Biased*, while most were in the *Accurate* group (see Table 2). The previously discussed phenomenon of persisting uncertainty towards the safety signal thus cannot be attributed to the participants’ occupational status. Still, conclusions may only be drawn with great caution and concerns of external validity need to be considered. Further, the classification of the three contingency awareness groups itself needs to be questioned, since there were no significant differences regarding temperamental traits or trajectories of reported fear towards both CSs. We did find *Threat Biased* participants to show persisting uncertainty towards the safety signal as reflected by significantly higher rated contingency probabilities. Future research trying to identify participants exhibiting this specific or similar patterns of bias, should aim to find markers that distinguish these individuals from those without such bias.

Knowing more about the underlying mechanisms connecting findings from fear conditioning research to clinically relevant phenomena is of great importance to the development of efficacious interventions. Fear conditioning as a translational model has informed treatment approaches to a great extent, however, there are large areas of uncertainty (Carpenter, Pinaire, & Hofmann, 2019). This illustrates the need to correctly link highly standardized laboratory evidence to real world phenomena. This branch of research may also help to identify individual’s predispositions to developing certain psychological disorders. Classifying such at-risk groups could subsequently help to recognize those who may benefit from early intervention or preventive measures (e.g., Stegmann et al., 2019). However, as Scheveneels, Boddez, and Hermans (2021) discussed, the necessary prospective research is still sparse and suffers from various problems regarding standardization and consistent between-study operationalization.

### Conclusions

This study aimed to test the feasibility of a differential fear conditioning paradigm, conducted on the home computer without a professional instructor present and introduced a unique way of calibrating an aversive sound. The procedure proved to be successful in inducing typical patterns of fear conditioning and extinction. Semi-subjective stimulus calibration, as introduced here, offers several advantages to researchers who try to implement fear conditioning paradigms outside of the laboratory context. However, we were not able to provide strong evidence that temperamental and personality traits can predict differences in fear learning.

## Data Availability

The datasets generated during and/or analyzed during the current study are available from the corresponding author on reasonable request.

## Appendix A: Semi-Subjective Stimulus Calibration Instructions

Original German instructions were translated into English. Instructions concerning the semi-subjective stimulus calibration were presented over the course of four PowerPoint slides.

**Slide 1:** In the following experiment, you will see various images. In addition, loud, unpleasant noises will be heard at unspecified intervals, which lie outside the range that is harmful to health. To avoid any health concerns, it is important to set the volume of your headphones correctly on the following slides. Please read the instructions carefully!

**Slide 2:** On the next slide you will hear the following sound in two different volumes:

***Displays a button with which to play the test beeping sound***

It is important that you adjust the overall volume, either directly on the headphones or in the settings of your PC! Do not change the volume within PowerPoint at this control

***Shows a picture of the volume slider from within PowerPoint with a red cross over it.***

**Slide 3:** Please listen to the following two sounds one after the other in a quiet environment:

Tone 1: Please adjust the volume so that you can only guess the first tone or can no longer hear it!

***Displays a button with which to play the softer test beeping sound***

Tone 2: The second tone should sound uncomfortably loud but not cause you any pain! If you do feel pain, adjust the volume slightly downwards so that it becomes bearable.

***Displays a button with which to play the louder test beeping sound***

Please listen to the two tones as often as necessary. Adjust the volume until the specifications are met.

**Slide 4:** If you have adjusted the volume of your headphones correctly, please take a pen and the documents sent to you by post. If you are unsure whether the volume is correct, go back one slide. Please remember that you have to change the overall volume and not the individual sound samples!

***Shows a picture of the volume slider from within PowerPoint with a red cross over it.***

## Declarations

### Preregistration

The study was preregistered using the *AsPredicted* preregistration form on OSF.io: https://osf.io/7bspk.
